# Supplementary material for: Leaf Functional Traits in Relation to Species Composition in an Arctic–Alpine Tundra Grassland
Source: Plants (Basel). 2023 Feb 22;12(5):1001. doi: 10.3390/plants12051001 (PMC10005651; doi:10.3390/plants12051001)
Supplement: Supplementary file 1 [file plants-12-01001-s001.zip › plants-2197676-supplementary.pdf]

Supplementary Table S1. Element composition of green leaves for studied species. Means and standard deviation (S.D.) for three collection dates during 2020. Different letters denote significant difference at α=0.05 according to one-way ANOVA and Kruskal-Wallis test.

**Element**		*C. villosa*	*D. cespitosa*	*M. caerulea*	*N. stricta*
**C (%)**	Mean	37.5	39.1	38.1	36.9
	S.D.	1.5	1.1	1.5	1.1
		bc	a	ab	cd
**Ca (mg/kg)**	Mean	1278	1372	1219	875
	S.D.	292	628	365	113
		a	a	a	b
**Mg (mg/kg)**	Mean	847	1372	1087	731
	S.D.	279	159	239	83
		bc	a	d	c
**K (mg/kg)**	Mean	21024	17096	15689	13470
	S.D.	6968	3889	3520	2264
		a	ab	bc	c
**N (%)**	Mean	24.7	29.2	28.2	20.6
	S.D.	7.8	6.1	6.6	4.0
		ab	a	a	b
**P (mg/kg)**	Mean	2122	3223	2031	1327
	S.D.	1027	655	713	320
		b	a	b	c
**C:N**	Mean	17.1	14.0	14.0	18.6
	S.D.	4.9	3.0	3.0	3.6
		ab	b	b	a
**N:P**	Mean	17.1	14.0	14.0	18.6
	S.D.	4.9	3.0	3.0	3.6
		ab	b	b	a
	n	18	12	18	18
